# Clinician‐Centric Explainable Artificial Intelligence Framework for Medical Imaging Diagnostics: A Systematic Review

**DOI:** 10.1155/ijbi/6366492

**Published:** 2026-04-24

**Authors:** Charles Ikerionwu, Ikenna Arungwa, Tochukwu Maduike Emelogu, Chidinma Esther Nwabuike, Elochukwu Ukwandu

**Affiliations:** ^1^ Department of Software Engineering, Federal University of Technology, Owerri, Imo State, Nigeria, futa.edu.ng; ^2^ Department of Surveying and Geoinformatics, Federal University of Technology, Owerri, Imo State, Nigeria, futa.edu.ng; ^3^ St. George Specialist Hospital, Effurun, Delta State, Nigeria; ^4^ Asokoro District Hospital, Abuja, Federal Capital Territory, Nigeria; ^5^ Department of Cyber Security, Cardiff School of Technologies, Cardiff Metropolitan University, Cardiff, Wales, UK, cardiffmet.ac.uk

**Keywords:** artificial intelligence, clinician-centric explainable AI, deep learning, explainable AI, machine learning, medical imaging

## Abstract

Medical imaging has evolved from conventional x‐rays to advanced digital modalities, with artificial intelligence (AI), particularly deep learning, showing an increasingly central role in diagnostic support. This study presents a systematic literature review (SLR) of AI‐driven medical imaging research focusing on classification‐based models and explainability approaches in pneumonia detection. Using predefined inclusion criteria and PRISMA‐guided screening, 95 studies were synthesized to identify dominant architectures, dataset trends, performance patterns, and persistent challenges. The analysis shows that convolutional neural networks (CNNs) and their variants remain the most frequently adopted models, accounting for the largest proportion of applications across x‐ray, computed tomography scan (CT scan), and magnetic resonance imaging (MRI). Reported diagnostic performance across reviewed studies commonly exceeded 90% in accuracy and AUC, with models such as DeepMediX, XNet, Wavelet‐CNN, and RadCLIP demonstrating strong predictive capability in their respective experimental settings. However, the review identifies significant gaps in explainability, clinical workflow integration, ethical compliance, and trust evaluation. Thus, this paper proposes a clinician‐centric explainable artificial intelligence (CC‐XAI) framework derived from literature synthesis. The framework integrates multilevel explainability, contextual clinical alignment, and human‐in‐the‐loop feedback mechanisms to bridge the gap between black‐box AI systems and real‐world clinical practice. Rather than introducing a new predictive model, the framework provides a structured design blueprint for embedding explainability into medical imaging diagnostics. The findings highlight the continued dominance of deep learning in medical imaging while emphasizing the urgent need for clinician‐oriented XAI frameworks to support transparency, trust, and responsible AI deployment in healthcare.

## 1. Introduction

Advances in medical image (MI) processing have transitioned significantly from conventional film‐based methods to advanced digital modalities that provide an unparalleled understanding of the human body. Since its discovery by Roentgen in 1895, MI has become a crucial diagnostic technique for various illnesses [1]. It has evolved into multiple modalities, such as computed tomography, ultrasound, magnetic resonance imaging (MRI), resonance imaging, and positron emission tomography. Image information is crucial in a patient′s care stages, like detection, staging, treatment response assessment, disease recurrence monitoring, and directing interventions. It has been recorded that the vital role of MI has contributed to patient care through quick decision‐making and precision diagnostics with high accuracy [2].

MI processing is widely used in healthcare due to its ability to extract features, analyze and interpret images, deliver high prediction accuracy, and support quick diagnoses [3]. Studies show that machine learning (ML) and deep learning (DL) significantly aid MI interpretation [4, 5]. However, the complexity and volume of image data remain challenging. Artificial intelligence (AI)—particularly ML and DL—helps reduce this burden while opening new possibilities in imaging. As noted in [6], AI‐driven tools enhance diagnostic accuracy, streamline workflows from data collection to analysis, and provide predictive insights. According to Khalifa and Albadawy [7], AI can analyze large volumes of visual data, identifying subtle patterns that humans may miss. This capability supports earlier and more accurate diagnoses, ultimately improving patient outcomes.

Extant literature has shown that AI can mechanize laborious and repetitive activities, such as picture segmentation and measurement, consequently improving prediction accuracy [8–10]. AI expedites the advancement of novel imaging methods and treatments by detecting patterns in extensive datasets and producing artificial images for training and verification. These allow radiologists and other healthcare professionals to allocate their time toward more intricate cases and patient care. By analyzing patient‐specific data, AI can assist in identifying the most effective treatment strategies and forecasting individual reactions to pharmaceutical interventions [6].

Although AI in MI came with fantastic promises, various problems abound, such as data quality and availability, algorithm transparency and explainability, and regulatory and ethical considerations [11]. Despite significant progress in AI‐based pneumonia detection from MIs, most existing models operate as black‐box systems, which provide limited or no clinically meaningful explanations. While high diagnostic accuracy is frequently reported, clinicians require additional transparency and reasoning that align with medical knowledge to support trust and decision‐making. The extant literature suggests that current explainable artificial intelligence (XAI) approaches are largely generic and algorithm‐centered and fail to integrate clinical workflows or address clinicians′ interpretability needs. Similarly, studies tend to emphasize performance metrics over explainability, usability, and clinical adoption. This gap underscores the need for a clinician‐centered framework that systematically bridges AI explainability and real‐world pneumonia diagnosis practice. Robust and extensive datasets are essential for training efficient AI systems. Safeguarding the accessibility of varied and inclusive data while simultaneously addressing privacy issues is paramount. AI models and intense learning networks can be intricate and lack transparency when developed haphazardly. Understanding how these models accomplish their goals is vital to establishing trust and ensuring safe and moral use in clinical practice. Integrating AI into MI poses significant challenges to safety, effectiveness, and responsibility [12, 13], hence defining the criteria and critical requirements for model development, testing, and validating the AI‐driven solutions model [14–16].

Earlier studies focused on traditional ML approaches for disease prediction. Larabi‐Marie‐Sainte et al. [17] reviewed several techniques, including decision trees, support vector machines, and neural networks for diabetes prediction. Their findings suggest that model performance depends strongly on feature selection and dataset quality. However, most of the applied models lacked transparency, making their decision processes difficult for clinicians to interpret. Similarly, research has shown that feature selection plays a critical role in handling large medical datasets. Shehu Aliyu et al. [18] proposed an enhanced feature selection approach for imbalanced microarray cancer classification using a chaotic salp swarm algorithm, demonstrating improved classification performance in high‐dimensional datasets. Despite these advances, selected features are often not presented in a clinically meaningful way, limiting their usefulness for clinical interpretation and decision‐making.

Similarly, Karimi et al. [19] presented a comprehensive survey of feature selection methods for big medical databases. The study highlighted improvements in model efficiency and accuracy through dimensionality reduction. Despite these benefits, the selected features are often not presented in a clinically meaningful way, thus limiting their usefulness for clinical interpretation and decision‐making.

Therefore, this study is aimed at performing a systematic literature review (SLR) to provide an in‐depth and breadth assessment of trends in MI underpinned by AI. Further, it will review the available research to uncover significant patterns, problems, and potential in this fast‐expanding topic. Further, the study seeks to evaluate how explainability methods are applied using measurable criteria such as transparency level, localization accuracy, and alignment with clinical reasoning. Based on the identified gaps, the study is aimed at deriving technical design requirements for clinician‐oriented explainability in pneumonia detection systems. Finally, the study proposes a clinician‐centric explainable artificial intelligence (CC‐XAI) framework, grounded in empirical evidence from the literature, to support interpretable, trustworthy, and clinically actionable AI‐assisted diagnosis. These findings will be essential in driving future research, development, and implementation of AI‐driven solutions, leading to better patient care and more efficient healthcare systems. To accomplish this objective, this study will conduct an SLR, meticulously analyzing prior research and the existing body of knowledge for AI‐powered advancements in MI.

### 1.1. Research Contributions

This study makes three key contributions. It presents a current and systematic synthesis of AI and XAI approaches for pneumonia detection—aligned to clinical usability rather than accuracy alone. It highlights critical limitations in existing explainability methods, especially trust, weak alignment with clinicians′ reasoning, and workflow needs. Finally, it introduces a CC‐XAI framework that provides a practical pathway for embedding explainability into real‐world pneumonia diagnosis to support trust, transparency, and clinical decision‐making.

In this study, CC‐XAI refers specifically to explainability mechanisms, which are tailored to clinical diagnostic reasoning, workflow integration, and decision support. This distinguishes it from general human‐centered AI, which addresses a broader range of users such as consumers, developers, and decision‐makers. Therefore, the scope of the study focuses on studies that cover AI algorithms, DL models, ML techniques, and their applications in MI modalities (e.g., MRI, CT, x‐ray, and ultrasound). This study does not involve training or evaluating a new AI model on a proprietary dataset; instead, dataset characteristics are analyzed comparatively across reviewed studies as part of the SLR. The next sections include disruptive technologies in MI, related works, methodology, SLR, analysis, findings, proposed CC‐XAI framework, and conclusion.

## 2. Disruptive Technologies in MI

Disruptive technologies represent groundbreaking innovations capable of fundamentally altering entire sectors by supplanting established systems, creating new markets, or rendering traditional methods outdated. They have advanced patient care, diagnosis, and therapeutic procedures in the medical field, with a particular effect on MI. Recent developments have markedly enhanced visualization and diagnosis through noninvasive methods, resulting in enhanced patient outcomes and a more streamlined healthcare delivery system [20]. According to extant literature, the research identifies AI, ML, and DL as three key technologies that have revolutionized MI [21–24].

**Figure 1:**
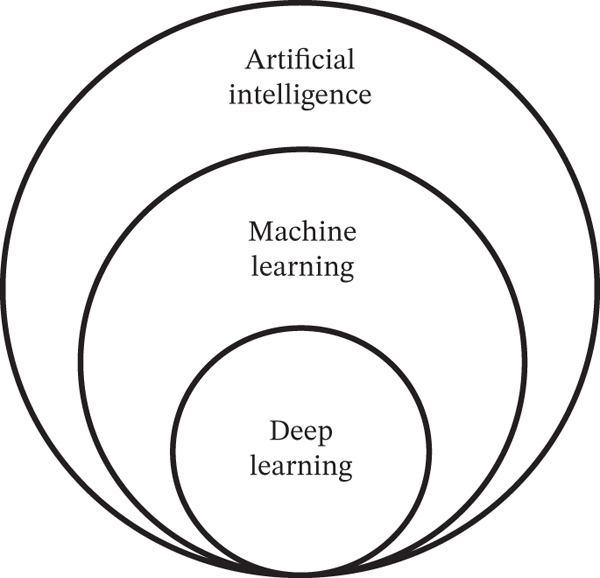
Disruptive technologies: AI, ML, and DL.

These are considered disruptive because they create new possibilities and opportunities and improve accuracy in diagnosis. Figure 1 introduces the relationship between AI, ML, and DL and specific benefits in MI.

Figure 1 is a hierarchical expression that denotes that all DL is ML, and all ML is AI. It implies that AI is the bigger picture, where ML is a subset of AI and DL is a subset of ML, which is mathematically expressed in Equation (1).

(1) DL⊂ML⊂AI

### 2.1. AI

**Figure 2:**
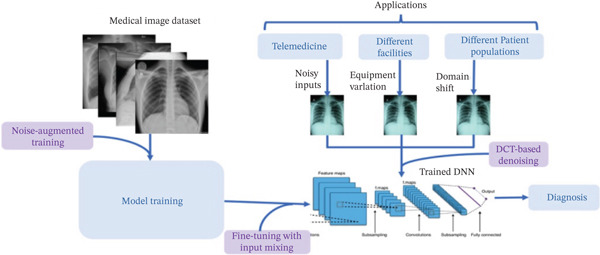
AI‐driven medical image processing workflow [30].

It is a broader field comprising a set of techniques and algorithms that create intelligent systems. Its application impact on MI reflects on the imaging process from data acquisition to diagnosis and treatment planning. Pinto‐Coelho [25] posits that the principal objectives of AI revolve around enhancing the capacities of healthcare professionals through the deployment of sophisticated algorithms and computational power to quickly and accurately process and analyze extensive quantities of image data, for example, the application of DL to predict the presence of a plasmodium parasite in a blood smear slide or the prediction of pneumonia from abnormal human lungs from 2000 image data [26, 27]. Different researchers have recorded consistent benefits that primarily improved clients′ healthcare and provided the background for further research. These include prediction accuracy, cancer detection, organ segmentation, automated diagnosis and reporting, efficiency and workflow optimization, remote diagnostics and teleradiology, early detection of disease, and radiomics [28, 29]. Figure 2 illustrates an AI deep neural network (DNN) training workflow for MI, emphasizing improving prediction accuracy.

The process begins with a pneumonia MI dataset for model training, data preprocessing, and augmentation, primarily to improve the models′ robustness and accuracy. Upon completion of the training, the DNN is deployed across different medical applications, such as telemedicine and disease diagnosis, to aid medical professionals in predicting different diseases or ailments [31]. The figure comprises the following process points that make up the workflow: The dataset is fed into the input section; the model training with add‐on services like noise augmentation and input mixing; fine‐tuning, which handles the variations in image quality; applications, which hold the device independence of the model; and the prediction outputs by DNN, aided by denoising and model robustness.

### 2.2. ML in MI

ML is a subset of AI, which is applied in MI for developing predictive models that can identify patterns from historical data (e.g., images and diagnoses)—its accuracy is seen to improve over time. Research shows that ML algorithms have been consistently applied in image classification, for example, differentiating benign and malignant tumors, regression algorithms in predicting the likelihood of diseases, and clustering—used in grouping similar image data. In Table 1, ML uses varied algorithms to perform different tasks in MI [42].

**Table 1:** ML algorithms in medical imaging.

Algorithm	Type	Application in medical imaging	Citation
Linear regression	Supervised	Used in predicting continuous outcomes (e.g., estimating disease progression and predicting tumor volume)	[32]
Logistic regression	Supervised	Classification tasks (e.g., detecting whether a tumor is benign or malignant)	[33]
Support vector machines (SVMs)	Supervised	Classifying images (e.g., distinguishing between healthy and abnormal tissue)	[34]
Random forest	Supervised	Image classification and disease detection (e.g., classifying MRI scans or x‐ray images)	[35]
K‐nearest neighbors (KNN)	Supervised	Image classification (e.g., classifying cells, tissues, or organs in images)	[36]
K‐means clustering	Unsupervised	Grouping similar patterns in images (e.g., detecting similar structures in MRI scans)	[37]
Principal component analysis (PCA)	Unsupervised	Dimensionality reduction and feature extraction from medical images (e.g., reducing complexity in radiology images)	[38, 39]
Autoencoders	Unsupervised	Image denoising, anomaly detection, and feature learning (e.g., reducing noise in medical images)	[40]
Decision trees	Supervised	Disease diagnosis and classification of medical images (e.g., classifying whether an image shows pathology or normal tissue)	[41]

Table 1 highlights different ML algorithms applied in MI in supervised and nonsupervised learning, focusing on classification, segmentation, and data augmentation.

### 2.3. DL in MI

DL is a specialized area within ML that uses neural networks with several layers to automatically learn high‐level features from images. According to extant research focusing on MI, DL models have been used extensively and effectively in handling complex image data such as x‐rays, MRIs, or CT scans. Specific tasks include image recognition, segmentation, and analysis of unstructured data. There are several types of models used in DL, such as a convolutional neural network (CNN), which has been extensively used in classifying MIs. In the work of [43], CNN has been demonstrated to autonomously extract complex patterns from raw images, which may not be easily identifiable by traditional methods. Table 2 presents DL models, their key characteristics, and specific tasks performed in MI processing. The CNNs are treated as a broad model category, with architectures such as fully convolutional networks (FCNs), VGGNet, ResNet, and 3D CNNs considered as specialized variants within this class. Models are listed separately to reflect how they are reported in the reviewed studies. To improve clarity, the models are categorized into architectural families, derived variants, and task‐specific implementations.

**Table 2:** DL models for medical imaging.

Model category	Model variant	Application	Citation
Core model	CNN	Classification and segmentation (e.g., tumor detection, organ segmentation, and detecting anomalies in radiology images)	[44]
CNN‐based model	ResNet	Classification and segmentation (e.g., detecting and diagnosing diseases like cancer in x‐rays and MRIs)	[45, 46]
	VGGNet	Classification and feature extraction in medical imaging (e.g., detecting abnormalities in images like x‐rays)	[47–49]
	DenseNet	Medical image classification and feature reuse	[50]
	InceptionNet	Classification and feature extraction (e.g., identifying and classifying diseases in medical scans)	[51, 52]
	Xception	High‐precision image classification in CT and MRI	[53]
Segmentation model (CNN‐based)	U‐Net	Segmentation (e.g., segmenting regions of interest such as organs or tumors)	[54]
	FCNs	Semantic segmentation of medical images (e.g., segmenting tumors or lesions from medical scans)	[55]
Segmentation model (3D CNN‐based)	3D CNN	3D medical image analysis (e.g., 3D volumetric data from CT and MRI scans)	[56]
	V‐Net	3D medical image segmentation (e.g., segmenting organs or tumors in 3D CT/MRI scans)	[56]
Segmentation model (attention‐based)	Attention U‐Net	Image segmentation, especially for small objects (e.g., segmenting small tumors or lesions in medical images)	[57]
Detection model (CNN‐based)	Faster R‐CNN	Object detection (e.g., detecting tumors or lesions in medical images)	[58]
	Mask R‐CNN	Instance segmentation (e.g., detecting and segmenting different objects or regions within medical images)	[59]
Generative model	GAN	Augmentation, generating synthetic medical images for training data	[60]
Sequential model	RNN	Analyzing sequential medical data (e.g., time‐series medical imaging data and patient monitoring)	[61, 62]
Lightweight model	MobileNetV2	Efficient and real‐time medical image classification	[63]
Representation learning model	Autoencoders	Feature extraction, dimensionality reduction, image denoising, and anomaly detection	[64]

Table 2 lists DL models that were utilized in MI for tasks such as data augmentation, segmentation, picture classification, and feature extraction. Most models are built on CNNs, which are frequently used for anomaly detection and image categorization. Specifically made for image segmentation, U‐Net and FCNs aid in tasks like organ or tumor segmentation. Utilizing deep architectures with residual or dense connections to improve performance, ResNet and DenseNet are used for feature extraction and classification. To get around the problem of sparse medical data, generative adversarial networks (GANs) are essential for data augmentation [65]. For the analysis of 3D medical data, including CT and MRI images, 3D CNNs and V‐Net are crucial because they offer sophisticated segmentation in volumetric images. Lastly, models such as Mask R‐CNN and Faster R‐CNN are designed for object recognition and segmentation, which improves diagnostic accuracy by recognizing and segmenting various areas or structures in medical pictures.

## 3. Review Method

**Figure 3:**
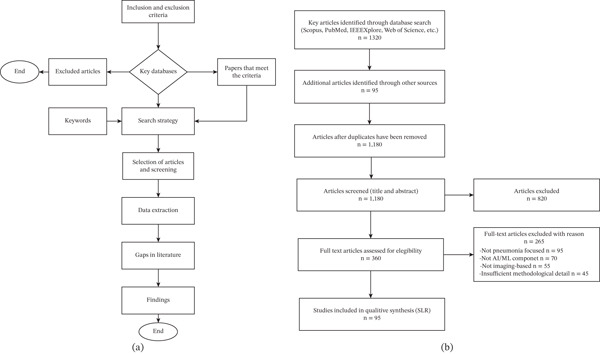
(a) Review methodology. (b) PRISMA flow diagram of study selection.

Figure 3a depicts the research methodology applied in this study. It focuses on different methods, techniques, tools, and procedures used in data collection, analysis, and interpretation. In designing the research methodology, the researchers considered the main objective and scope of this study as outlined in the Introduction section. In the SLR, the researchers first established the inclusion and exclusion criteria to be applied to the database search.

Figure 3a illustrates the steps involved in the SLR process, while Figure 3b presents the PRISMA flow diagram with the number of studies included and excluded at each stage.

a. *The inclusion criteria*: These were applied to the selected databases to identify relevant articles. It stipulated the review to include only peer‐reviewed journal articles, conference papers, and high‐quality academic publications to ensure credibility and reliability. To reflect current advancements in AI and MI, only articles published within the last 10 years were considered. Additionally, the selection criteria prioritized studies emphasizing AI techniques such as DL, ML, and AI‐driven diagnostic systems in MI. Research detailing the results of AI applications in specific MI modalities was also included to provide a comprehensive understanding of the field.
b. *The exclusion criteria*: These criteria were applied to ensure that papers not related to the study were excluded from the search. This implies that all studies not related to MI or AI applications in healthcare are excluded. Similarly, gray literature and non‐peer‐reviewed papers are not considered unless they contribute significant, unique outcomes. Also, articles that do not address diagnostic applications but are purely technical AI studies without a medical focus were excluded.
c. *Identifying key databases for the literature search*: First, the researchers identified the PubMed database for the listing of biomedical and clinical research related to MI and AI diagnostics and, similarly, IEEE Xplore for articles on AI algorithms, ML, DL, and applied MI. Google Scholar was selected to provide a wide range of academic papers, theses, and multidisciplinary reports on MI. Scopus provides comprehensive citation data, including journal articles, conference proceedings, and patents. Web of Science and ScienceDirect were shortlisted to provide highly cited papers, review articles across a broad range of scientific disciplines, and medical and healthcare journals that focus on AI in MI.
d. *Search strategy*: To ensure comprehensive coverage of the search, the researchers used a combination of specific and broad keywords. Only studies published between 2016 and 2025 and written in English were included in the review. First, primary keywords include “AI in medical imaging,” “artificial intelligence diagnostics,” “machine learning in radiology,” “medical imaging Deep learning,” “deep learning for image analysis,” “medical imaging breakthroughs AI,” and “AI in MRI/CT/X‐ray.” The Boolean operators were used to combine two or more terms: AND, OR, and NOT operators, for example, “artificial intelligence AND medical imaging AND diagnostic accuracy AND deep learning,” “AI AND cancer detection AND MRI AND deep learning,” and “machine learning AND medical image segmentation AND radiology.” To reduce irrelevant retrievals, wildcard terms (e.g., medical∗) were combined with domain‐specific keywords, while controlled vocabularies such as MeSH terms were used for structured searches in PubMed such as (“Artificial Intelligence”[MeSH] OR “Machine Learning”[MeSH]) AND (“Pneumonia”[MeSH]) AND (“Diagnostic Imaging”[MeSH]), “medical∗”, among others.
e. *Screening and selection of articles*: The article selection process involved four stages: initial screening of titles and abstracts for relevance, full‐text review based on inclusion criteria, exclusion of studies unrelated to AI in MI or diagnostics, and a final quality assessment considering peer review status, journal impact factor, citation count, and methodological rigor.
f. *Data extraction*: Key information extracted includes citation, study aim, dataset type, ML model used, and key diagnostic findings. These elements provide a structured basis for comparing methodologies and outcomes across the reviewed literature.
g. *Data analysis visualization*: Python programming language and its rich libraries were used for the analysis and data visualization for all the key findings. Visualization tools include tables, graphs, and bar charts.
h. *Gaps in the literature*: The gaps in the literature are identified through a detailed SLR that presents the areas for further research.
i. *PRISMA flowchart*: The PRISMA flowchart, as depicted in Figure 3b, illustrates the study selection process in a structured and transparent manner. A total of 1320 records were identified through database searches, while 95 additional records were obtained from other sources, such as Google Scholar and complementary sources. After removing duplicates using the inclusion and exclusion criteria, 1180 unique records remained for title and abstract screening. Here, 820 records were excluded for not meeting the inclusion criteria. The full texts of the remaining 360 articles were assessed for eligibility, and 265 articles were further excluded based on predefined criteria. Finally, 95 studies satisfied all inclusion requirements and were included in the final synthesis of the review.

## 4. SLR

The SLR focused on five thematic areas: citations, aim, dataset, types of AI models, and research findings. Each of these areas synthesizes the key requirements in the theme.

Current literature suggests that the recent state‐of‐the‐art approaches, including transformer‐based architectures, hybrid CNN‐attention models, and post hoc explainability techniques such as Grad‐CAM++, SHapley Additive exPlanations (SHAP), and LIME, are integrated in pneumonia detection models [82]. Thus, they are explicitly analyzed along with related algorithms in the SLR that informed the design principles of the proposed framework.

## 5. Analysis of the SLR

**Figure 4:**
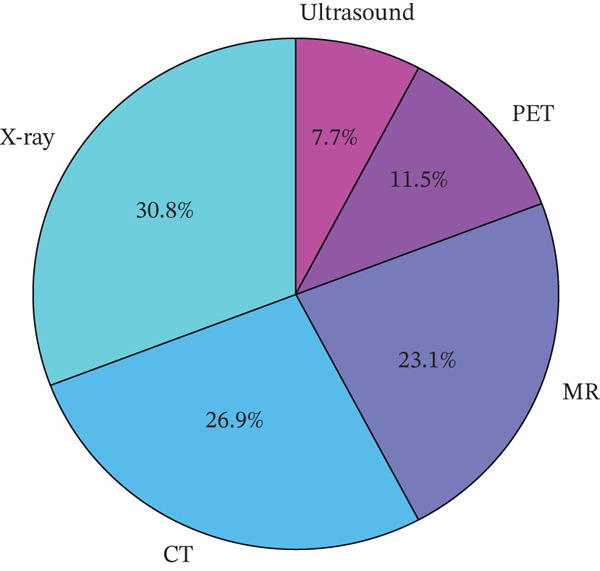
Distribution of AI applications across medical imaging.

This section presents the analysis performed on the SLR to bring out the salient features in the data. Figure 4 shows the distribution of AI applications in MI using different models, while Table 3 provides the trends. There are noticeable MI applications in x‐ray, ultrasound, PET, CT scan, and MRI. Respectively, 30.8% of the x‐rays performed were applied through AI in MI, 7.7% were performed in ultrasound, 11.5% in PET, 23.1% in MRI, and 26.9% in CT scan. These show the predominant growth of AI applications in MI.

### 5.1. Strengths and Weaknesses of AI Algorithms

Although DL has revolutionized MI techniques by reducing computational cost and improving accuracy and prediction, its variants have come with varied strengths and weaknesses.

Table 4 presents the strengths and weaknesses of AI algorithms within MI diagnostics. Works have shown that GAN has greatly improved data augmentation, but its computational cost is high and poses challenges in model stability [1]. CNN and its variants emerged as the dominant DL architecture with noticeable advancements such as transfer learning, data augmentation, and cross‐modal learning. XAI is another significant advancement that made DL models more transparent and interpretable. Each of the listed algorithms in Table 4 has demonstrated key strengths and weaknesses, but the overwhelming side has consistently made the algorithm stand out.

### 5.2. Evaluation of Performance AI Models

**Figure 5:**
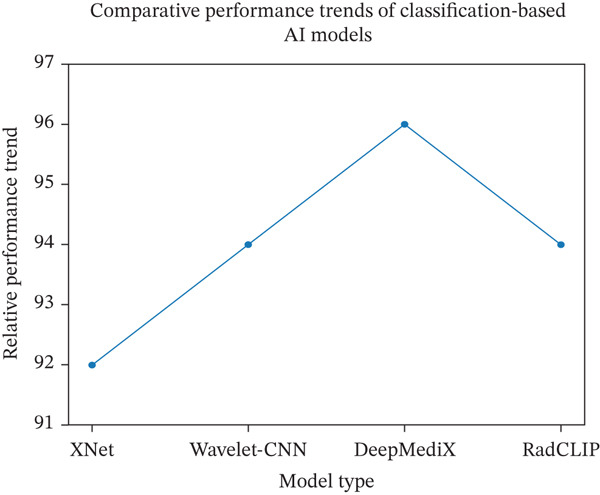
Performance trends of AI classification models in medical imaging.

Figure 5 presents a comparison derived from Table 3 as reported trends in the reviewed literature and does not represent statistical aggregation from a single dataset or experimental benchmark. It is a line graph that shows the comparison of these models synthesized from SLR. The line graph illustrates performances by AI models for MI based on three key indicators: accuracy, *F*1 score, and AUC. Generally, all models performed well, showing metrics above 90%, which demonstrates the effectiveness of AI in MI. However, DeepMediX has the highest accuracy and *F*1 score, confirming its suitability for real‐time predictions and efficient use of resources.

**Table 3:** Systematic literature review.

Reference ID	Aim	Dataset	Types of AI models	Findings
[66]	To develop a convolutional neural network (CNN) architecture, called XNet, for the segmentation of medical x‐ray images	69 CT scan images of feet, knees, and phantom heads Eighty‐one standard x‐ray images of different body parts	XNet (convolutional neural network)	92% accuracy, 0.92 *F*1 score, and 0.98 AUC outperforming classical methods and existing networks
[67]	To provide an overview of deep learning applications in medical image analysis	Medical imaging datasets such as MRI, CT, x‐ray, PET, and fMRI	Various deep learning algorithms, including CNN, RNN, and LSTM	Deep learning has greatly improved accuracy and efficiency in medical image analysis
[68]	It aims to propose a deep learning–based framework for medical image classification using wavelet features	X‐ray images from the ImageCLEFmed 2005 dataset	Haar wavelet, LeNet‐based CNN for feature extraction	The wavelet‐based CNN improves x‐ray classification with higher accuracy and lower computational cost
[69]	It aims to provide a detailed review of transfer learning in medical image analysis	CT scans, MRI scans, ultrasound images, and x‐rays	CNN and its variants	The paper highlights transfer learning′s role in speeding up and reducing the cost of medical image model training
[21]	To overview of AI in medical imaging, highlighting key ML and DL methods	CT scans and MRI scans	Random forests, CNN, and GAN	Promotes medicine–computer science collaboration for robust, interpretable clinical AI
[70]	Aimed to provide explainable artificial intelligence (XAI) techniques in DL MI analysis	CT scan and x‐ray	LeNet, VGG‐16, and VGG‐19	The findings categorized XAI techniques based on a proposed framework
[3]	Develop a machine learning framework to enhance medical image diagnosis	CNN	Medical images (MRI, NMR, PET, SPECT, CT, and ultrasonography)	The framework improves CNN‐based diagnosis via image processing steps and supports expert system development
[71]	Aimed at implementing deep learning in medical image processing	DCNN, CNN, RNN, LSTM, SVM, DBN, and autoencoders	Medical images (x‐ray, CT, and MRI)	Deep learning automates medical image analysis but struggles with data scarcity and low interpretability
[72]	To develop and validate a deep learning CAD system to aid radiologists in enhancing diagnostic accuracy and workflow	X‐rays, computed tomography (CT) scans, and magnetic resonance imaging (MRI)	Deep convolutional neural networks (DCNNs)	The deep learning–based CAD systems exceeded conventional methods and human experts in some tasks
[33]	A review of deep learning–based medical image segmentation methods	CT, MRI, PET, x‐ray, and ultrasound imaging	CNNs, FCNs, U‐Net, and GANs	Improved deep learning medical image segmentation and significant advancements
[73]	To survey deep learning–based medical image registration, key challenges, and future directions	CT, MRI, US, and x‐ray	CNNs and GANs	Demonstrates that deep learning–based methods outperform traditional optimization–based techniques
[74]	Evaluate the effect of deep learning–based methods in radiology image reconstruction and enhancement and compare it with traditional methods	Public radiology image datasets (LIDC‐IDRI for lung CT images and IXI for magnetic resonance images)	CNN, GAN, and autoencoder‐based imaging networks	Deep learning models, particularly GAN, outperformed traditional image quality, noise removal, and image detail restoration methods
[75]	Detect GAN‐based minor region forgeries in medical images	Medical images (primarily CT scans in DICOM format)	Two‐stage cascade with local detection with GLCM‐PCA‐based global classification	Superior performance compared to state‐of‐the‐art methods in detecting GAN‐based minor region forgeries in MI
[76]	To detect and diagnose breast cancer using AI models	Breast x‐ray mammography	CNN, SVM, and RF	Accuracy rates up to 99%, with enhanced sensitivity and specificity for tumor detection
[77]	Enhance the performance of AI and deep learning models by addressing the challenge of limited medical imaging data availability	Malignant, benign, or regular brain images from the BraTS20 dataset	GAN and variational autoencoder (VAE)	GAN and Disc‐VAE data augmentation notably boost AI classification accuracy over nonaugmented models
[78]	To examine the transformative impact of AI on medical imaging technology and its evolution	A detailed review	Discussed various algorithms used in different studies	AI improved medical diagnosis through medical imaging and treatment
[79]	To explore ML and DL in medical imaging, focusing on efficiency, complexity, interpretability, and scalability	A review paper	Focused on ML and DL algorithms applied in medical image analysis	Findings show AI has advanced medical image analysis, but efficiency, complexity, and scalability remain key for real‐world use
[80]	To examine how deep learning enhances medical image analysis for better diagnosis and care	A review paper	Discusses deep learning algorithms: CNN and RNNs	DL algorithms significantly enhanced medical image analysis and substantial healthcare outcomes
[81]	To develop a resource‐efficient deep learning model, DeepMediX, for classifying brain MRI scans and skin cancer images	ISIC2018 for dermatological research	MobileNetV2 architecture and federated learning	DeepMediX showed exceptional diagnostic capabilities, outperforming existing models. Solved lightweight computation and real‐time prediction
[82]	To automate the justification analysis of brain CT referrals and compare model performance to human experts	2958 anonymized brain CT referrals (2020–2021): justified, potentially justified, or unjustified	Gradient boosting (BoW), Bi‐LSTM, MLP, and SVM	Gradient boosting classifier achieved 94.4% accuracy and a macro *F*1 score of 0.94, outperforming DL models (Bi‐LSTM: 92.3% accuracy)
[83]	To evaluate AI tools for detection, classification, segmentation, and radiomics in medical imaging	Varied datasets including MRI, CT, x‐rays, and histological data	CNNs and radiomics‐based ML models	AI tools enhance diagnostic accuracy and efficiency, often outperforming humans in specific tasks
[84]	To review CNN applications in medical imaging, including advances, challenges, and future directions	BRATS (2013, 2015), CT scans for interstitial lung diseases, and IRMA	CNN (U‐Net, V‐Net, and 3D)	CNNs outperform traditional methods in segmentation, classification, detection, and retrieval tasks, with improved accuracy
[85]	RadCLIP applies cross‐modal CLIP to enhance radiologic image analysis	Comprehensive and diverse dataset of radiologic image–text pairs	Novel 3D slice pooling and CLIP for aligning images with text annotations	RadCLIP aligns radiological images with text and provides a strong vision backbone
[86]	To detect COVID‐19 using chest x‐ray images through neural network techniques	Chest x‐ray images	Enhanced neural network (ENN)	Achieved high accuracy (up to 98.99%) using optimized training strategies

Figure 5 is synthesized from the reviewed studies summarized in Table 3. It presents performance trends across selected classification‐based models for pneumonia detection, including XNet, Wavelet‐CNN, DeepMediX, and RadCLIP. The figure does not represent a unified benchmark but reflects patterns reported in different studies. From the figure, DeepMediX is associated with stronger reported outcomes, while Wavelet‐CNN and RadCLIP show competitive performance. XNet also demonstrates consistent diagnostic capability within its respective studies. Because the models were evaluated on different datasets and validation settings, the trends are indicative and not direct statistical comparisons among models. Thus, this synthesis highlights ongoing progress in classification‐based AI approaches for MI.

### 5.3. Advances in AI for MI

**Figure 6:**
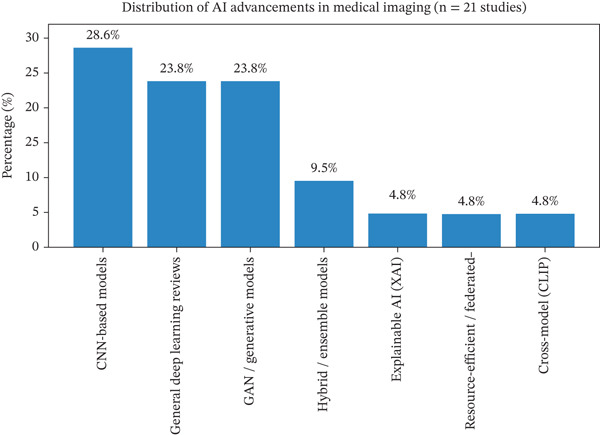
Distribution of AI advancements in medical imaging.

Figure 6 shows the distribution of AI advancement themes identified from the included studies (*n* = 21). The percentages were calculated based on the number of studies that used each AI approach as their primary method, relative to the total number of included studies. The chart shows that CNN‐based models constitute the largest proportion of approaches, followed by general DL frameworks and GAN‐based methods. Hybrid models, XAI, resource‐efficient techniques, and cross‐modal approaches appear less frequently. The figure highlights the dominant methodological trends in AI‐driven MI research for pneumonia detection. The percentage is calculated as the proportion of studies per category relative to the total included studies (*n* = 21).

Percentage=model count21×100

where model count is the number of included studies (*n* = 21) that used a specific AI approach as their main method, as presented in Table 3.

Several studies [87, 88] reported 99% prediction accuracy in AI‐based MI. Van der Velden et al. [70] highlight XAI frameworks as key to making diagnostics interpretable and trustworthy. Data availability, enhanced through augmentation, and improvements in model efficiency and computational complexity have also contributed significantly to recent advancements [89, 90].

### 5.4. Popularity of Algorithms in MI

**Figure 7:**
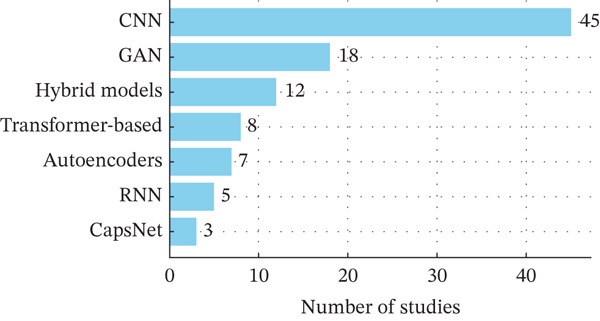
Algorithm frequency in medical imaging studies.

The number of studies in the SLR presented in Table 4 indicates that an increasing number of CNN algorithms are being used in radiology compared to other algorithms. Figure 7 shows the comparison between CNN and 20 other algorithms applied in various MI research. Li et al. [80] posit that CNN and its variant perform better than other algorithms in MI, such as x‐rays, CT scans, and MRIs.

**Table 4:** Strengths and weaknesses of AI algorithms in medical imaging.

Algorithm	Strengths	Weaknesses
GAN	Effective for generating high‐quality synthetic data and improving data augmentation.	High computational cost and challenges with model stability and interpretability.
CNN	Highly accurate in classification and segmentation; widely adopted.	Requires a large, labeled dataset; less effective with small datasets.
RNN	Good for sequential and time‐series data analysis.	Not optimal for static medical images; better for temporal data.
LSTM	Can handle long‐term dependencies in temporal medical data.	Computationally expensive; not ideal for static image analysis.
Wavelet‐based CNN	Enhances classification accuracy with reduced cost using wavelet features.	Limited generalizability; depends on wavelet feature quality.
MobileNetV2	Lightweight and efficient; suitable for mobile and low‐power devices.	Limited performance in highly complex tasks.
RadCLIP	Integrates radiology images and text for improved context.	Requires diverse datasets; high computational training cost.

CNN dominance is attributed to its nature of scalability and advanced technologies such as XAI and federated learning [91]. This is followed by GANs and hybrid models, which reflect their adaptability in classification, detection, and segmentation tasks.

### 5.5. Gaps in the Literature

Considering XAI′s contributions to MI, there remains a lack of frameworks that effectively bridge black‐box AI models and end‐users like radiologists. Limited exploration exists in handling diverse and sparse datasets, despite the use of federated learning and multimodal approaches. While Van der Velden et al. [70] discuss XAI in detail, it fails to address ethical standards crucial for clinical decision‐making. Research largely emphasizes technical metrics like accuracy and AUC, with insufficient attention to explainability and usability in real‐world settings. Additionally, studies on lightweight, scalable models balancing performance and efficiency are limited. Thus, robust frameworks enabling cross‐modality and cross‐dataset generalization are also lacking but hold great potential for improving model applicability.

## 6. Findings

This section presents the findings from the analysis performed on the systematic review and highlights the key contributions, such as performance metrics, challenges, and opportunities provided in AI MI. Table 5 reveals seven specific areas of focus from the analysis of SLR. These include integration of AI into MI, noticeable advances recorded in the AI algorithms, standard performance metrics, explainable challenges, data limitations, challenges from ethical and regulatory issues, data scalability, and real‐time applications.

**Table 5:** Key findings.

Key findings	Highlights
AI integration in medical imaging	There are significant advancements in diagnostic accuracy and efficiency across MRI, CT, x‐ray, and PET modalities.
Advancements in algorithms	CNN dominance in tasks like classification and segmentation; variants like U‐Net and GANs excel in other areas.
Performance metrics	There are high metrics, e.g., prediction accuracy of 99%, and 90% and above recording of AUC in MI, respectively.
Explainability challenges	Lack of interpretability in black‐box models and the proposed XAI frameworks to improve transparency.
Data limitations	GANs and VAEs used for synthetic data generation to address scarcity and heterogeneity issues.
Ethical and regulatory issues	Challenges with data privacy, transparency, and compliance (e.g., GDPR and HIPAA) addressed by ethical frameworks.
Trends in AI imaging	Recent trends highlight sophisticated innovations such as 3D and 4D imaging, real‐time disease prediction, and teleradiology.
Scalability and real‐time applications	Lightweight models like DeepMediX enable resource‐efficient and real‐time diagnostics.

These findings provide the current state of AI in MI, advances, strengths, challenges, and future directions.

### 6.1. Proposed CC‐XAI Framework

**Figure 8:**
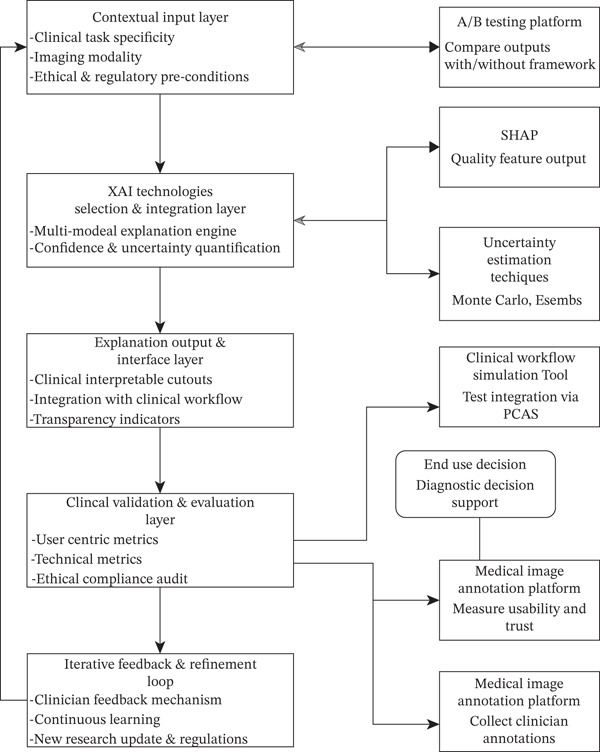
Clinician‐centric XAI framework.

Considering the critical gaps outlined in the SLR and in Table 5, which highlighted a lack of trust and interpretability hindering the full potential of AI in MI, this paper proposes the CC‐XAI framework to mitigate the hindrances. This framework is designed to bridge the divide between complex AI models that lack transparency in decision‐making and the practical needs of end‐users, such as radiologists and clinicians, within the context of the healthcare system. The clinicians are at the core of the explainability process, and the CC‐XAI framework emphasizes the delivery of explanations that are not only technically sound but also clinically relevant, understandable, and actionable. In Figure 8, the framework aims to foster greater adoption and effective utilization of AI diagnostic tools by enhancing their trustworthiness and utility in real‐world clinical practice.

Figure 8 illustrates the proposed CC‐XAI framework, which integrates explainability, clinician feedback, and diagnostic workflows, thereby bridging the gap between AI model outputs and practical clinical decision‐making. In the table, the CC‐XAI framework comprises four key layers and two sublayers, respectively:

i. Contextual input layer

The contextual layer gathers relevant diagnostic details to generate tailored explanations, considering the clinical task, imaging modality (e.g., x‐ray and CT), clinician′s role, and AI model characteristics. It also integrates ethical guidelines and regulatory standards to ensure the XAI process is transparent, appropriate, and responsible from the start.

ii. XAI technologies (selection and integration)

By using the contextual inputs, this layer is responsible for intelligently choosing the most suitable XAI techniques to generate insights. It houses a diverse toolkit of XAI methods, ranging from those that highlight important image regions (local explanations) to those that explain overall model behavior (global explanations) or provide “what‐if” scenarios (counterfactuals). The selection process aims to match the chosen techniques with the AI model′s architecture and the specific explanatory needs identified in the contextual input layer. This process reinforces users′ confidence and quantifies uncertainty when it arises.

iii. Explanation output and interface layer

This layer converts complex XAI outputs into clear, actionable formats for clinicians using intuitive visuals or plain language. It avoids technical jargon and integrates seamlessly with clinical systems like picture archiving and communication system (PACS) and electronic medical records. It also promotes transparency by indicating prediction confidence and known limitations of the explanation method, ensuring usability within existing clinical workflows.

#### 6.1.1. End‐User Decision

The clinician, as the end‐user, utilizes the AI′s prediction along with the tailored explanation provided by the CC‐XAI framework to inform their diagnostic decision‐making. It is noted that the framework ensures the explanation aids, rather than replaces, the clinician′s expertise and final diagnostic responsibility.

iv. Clinical validation and evaluation layer

The critical layer evaluates the utility, trustworthiness, and effectiveness of XAI outputs in clinical settings. It combines technical metrics with clinician feedback on explanation clarity and usefulness while also evaluating explanation fidelity and robustness. An ethical compliance audit ensures alignment with medical ethics and healthcare regulations, reinforcing the reliability of the XAI system.

#### 6.1.2. Iterative Feedback and Refinement Loop

This stage provides a continuous loop that drives the CC‐XAI framework′s evolution by systematically collecting and analyzing feedback from clinical applications and direct clinician input on explanation utility. This gathered information is then used to refine all aspects of the framework, from contextual parameters and XAI technique selection to explanation presentation and evaluation metrics. The iterative process ensures the framework remains adaptive to new AI models, emerging XAI methods, evolving clinical needs, and changing ethical or regulatory landscapes.

To ascertain the efficacy of the framework, four external modules are integrated into the framework for the following reasons:

i. A/B testing module: compares the decision accuracy with and without the framework.
ii. SHAP and uncertainty estimation module: provide a depth and breadth understanding of the model behavior.
iii. Workflow simulation tool: tests how the framework fits into a clinical process using synthetic cases.
iv. MI annotation platform: collects annotations for training/refinement and measures trust and usability.

### 6.2. Evaluation of CC‐XAI Framework With Related Works

To demonstrate its novelty, the CC‐XAI framework was compared with existing XAI approaches, including Van der Velden et al. [70], Ribeiro et al.′s LIME [90], and Lundberg and Lee′s SHAP [91]. Literature suggests that related frameworks were developed by [70, 90], which provide a comprehensive survey and classification of existing XAI techniques within MI, though none focused on CC‐XAI. Table 6 narrows the evaluation of [70] to the proposed CC‐XAI framework. While their work offers a foundational taxonomy of available methods, this paper proposes the CC‐XAI framework to specifically address operational, ethical, and usability gaps pertinent to clinical end‐users, as identified in our systematic review. The table offers a comparative analysis to delineate the distinct objectives, focus areas, and contributions of both the CC‐XAI framework and the work by [70].

**Table 6:** CC‐XAI framework versus Van der Velden et al. [70]—A feature comparison.

Feature	Van der Velden et al. [70]	Proposed CC‐XAI framework
Clinical task specification	✓	✓ (extended)
Regulatory compliance handling	✗	✓
Explanation clarity	Partial	✓ (clinician‐rated)
Trust evaluation	✗	✓
Workflow integration	✗	✓
Iterative improvement and adaptability	✗	✓
Clinician audience	✗	✓

Table 6 is a comparative analysis between the proposed CC‐XAI framework and the significant work on XAI techniques by [70]. The comparison highlights fundamental differences in their primary purpose, operational nature, and key focus areas within the domain of XAI in MI. It specifically contrasts their approaches to clinician‐centric design, integration of ethical standards for clinical decision‐making, emphasis on user‐centric evaluation, and suitability for practical application in healthcare environments.

Unlike Van der Velden, which focuses primarily on feature transparency, CC‐XAI extends explainability by integrating a clinician‐feedback loop, ensuring that explanations are not only algorithmically sound but also clinically meaningful. In contrast to LIME and SHAP, which provide post hoc model interpretability, CC‐XAI embeds interpretability throughout the workflow, from data preprocessing to diagnostic output. Moreover, while most prior frameworks treat clinicians as passive recipients of AI outputs, CC‐XAI explicitly positions them as active collaborators, thereby enhancing trust, accountability, and adoption in clinical settings.

### 6.3. Literature Grounding of the CC‐XAI Framework

This section demonstrates how the proposed CC‐XAI framework is grounded in evidence synthesized from the systematic review. Each component of the framework was derived from patterns and gaps identified in the analyzed studies. The framework reflects periodic methodological trends observed in prior research. The mapping presented in Table 7 establishes a link between the reviewed literature and the structural design of CC‐XAI.

**Table 7:** Mapping of the CC‐XAI framework to the reviewed literature.

CC‐XAI component	Supporting studies (cited in Table 3)	Observed gap in the literature	Design rationale
Diagnostic AI core	[66, 68, 72, 76, 82, 83, 85]	Strong predictive performance reported but focus largely on accuracy rather than interpretability	Retains high‐performing architectures while embedding structured explainability mechanisms
Multilevel explainability layer	[68, 70, 76]	Explanations often limited to saliency maps or visual heatmaps without semantic reasoning	Introduces layered explanations, including visual, feature‐level, case‐based similarity, and uncertainty reporting
Clinical context integration layer	[67, 78–80]	Limited integration of radiological reasoning or clinical workflow alignment	Embeds diagnostic reasoning patterns and contextual interpretation in the explainability process
Trust and feedback loop	[81–83, 92–96]	Minimal support for clinician override, feedback integration, or confidence calibration	Includes human‐in‐the‐loop mechanisms, confidence calibration

Table 7 illustrates how the CC‐XAI framework is directly informed by the reviewed studies. The Diagnostic AI Core is grounded in high‐performing CNN and hybrid models that dominate the literature. The multilevel explainability layer responds to the common reliance on basic saliency maps by incorporating broader explanation strategies. The clinical context integration component addresses the limited alignment between AI outputs and radiologists′ reasoning processes. Finally, the trust and feedback mechanism reflects the need for human oversight and confidence calibration identified across existing works. Conclusively, the table shows that the CC‐XAI framework is a structured response to documented gaps in the literature and is firmly grounded in existing evidence.

### 6.4. Conclusion

This paper takes a substantial step by proposing the CC‐XAI framework as a structured solution to the challenges identified in the literature. Thus, the framework is designed to bridge the critical gap between the “black‐box” nature of many advanced AI models and the practical, day‐to‐day requirements of clinical end‐users. Its core principle of clinician‐centricity ensures that explanations are tailored to the specific diagnostic task and user role clinician workflow. The CC‐XAI framework integrates ethical considerations, clinician‐centered design principles, and structured feedback mechanisms into the explainability process. It is designed to promote transparency and support clinically meaningful AI‐assisted decision‐making.

The proposed CC‐XAI framework is derived from a systematic synthesis of existing research. Compared to the traditional black‐box models, the framework integrates explainable outputs that align with clinicians′ diagnostic reasoning. The framework addresses key limitations in current XAI approaches by emphasizing interpretability, trust, and alignment with clinician workflow. Rather than focusing solely on model performance, the design prioritizes meaningful integration into real diagnostic workflows. The structure provides a practical blueprint for incorporating explainability into MI systems.

The findings from the SLR show the transformative potential of AI in MI, with particular attention on solving the long‐standing challenges of explainability, ethical integration, dataset scarcity, and scalability. Similarly, the findings underscore the urgent need for a pluralistic approach to AI‐driven MI that integrates scalability, interpretability, ethical integrity, and prediction accuracy. Disruptive technologies, including ML and DL, have led to groundbreaking innovations in MI. As a result of these significant advances in AI algorithms, CNN has performed better than the traditional methods used in tasks such as segmentation, classification, and outlier detection. Among the present algorithms, CNN and its variants have dominated MI in x‐rays, followed by CT scans and MRI. Noticeable advancements in MI include enhanced prediction accuracy, improved model efficiency, advanced data augmentation, greater model explainability, and reduced computational complexity.

A comparative synthesis of DeepMediX, XNet, Wavelet‐CNN, and RadCLIP, based on reported *F*1 score, accuracy, and AUC values in the reviewed studies, suggests that DeepMediX is associated with relatively stronger performance trends. It shows the superior performance and better generalization exhibited by DeepMediX in all the performance metrics. Considering the areas of AI applications, Figure 4 indicates that there are more applications of AI in x‐ray imaging than in other areas such as CT scan, MRI, PET, and ultrasound. The study identified the challenges of explainability emanating from the lack of interpretability in predictive models.

Although CNN‐based models have demonstrated strong diagnostic performance, their black‐box nature has limited clinical trust and adoption. Similarly, GAN‐based approaches, while useful for data augmentation, also lack inherent interpretability. Consequently, this study suggests a tailored XAI framework to ensure an understanding and quick interpretation of model predictions. Besides these advances and findings, the literature shows that while transfer learning and federated approaches have addressed data heterogeneity to some extent, the lack of standardized datasets and low interpretability in predictive models remain key challenges requiring further study.

The implementation of the CC‐XAI framework in a real‐world context is identified as future work. The focus will be on deploying the proposed framework through a phased approach. The first phase involves prototype development by integrating the framework with existing DL models and MI datasets. This will be followed by controlled validation using retrospective datasets and simulated clinical workflows to evaluate interpretability and usability. The third phase focuses on pilot testing in clinical settings with radiologists to evaluate trust, diagnostic effectiveness, and workflow integration. Finally, full‐scale deployment will integrate the framework into hospital systems such as PACS and electronic medical records, with continuous monitoring and refinement based on clinician feedback and regulations.

## Funding

The researchers acknowledge the support of the Nigeria Artificial Intelligence Research Scheme (NAIRS), in collaboration with the National Information Technology Development Agency (NITDA), under Grant No. NITDA/HQ/RG/AI9204249605.

## Conflicts of Interest

The authors declare no conflicts of interest.

## Data Availability

The data that support the findings of this study are available upon request from the corresponding author. The data are not publicly available due to privacy or ethical restrictions.
